# Time-resolved momentum microscopy with fs-XUV photons at high repetition rates with flexible energy and time resolution

**DOI:** 10.1038/s41598-025-86660-1

**Published:** 2025-01-29

**Authors:** Karl Jakob Schiller, Lasse Sternemann, Matija Stupar, Alan Omar, Martin Hoffmann, Jonah Elias Nitschke, Valentin Mischke, David Maximilian Janas, Stefano Ponzoni, Giovanni Zamborlini, Clara Jody Saraceno, Mirko Cinchetti

**Affiliations:** 1 Department of Physics, TU Dortmund University, Otto-Hahn-Straße 4, 44227 Dortmund, Germany; 2 Photonics and Ultrafast Laser Science, Ruhr University Bochum, Universitätsstrasse 150, 44801 Bochum, Germany; 3 Present Address: Laboratoire des Solides Irradiés, CEA/DRF/lRAMIS, Ecole Polytechnique, CNRS, Institut Polytechnique de Paris, Palaiseau, F-91128 France; 4 Present Address: Institute of Physics, NAWI Graz, University of Graz, Universitätsplatz 5, 8010 Graz, Austria

**Keywords:** Techniques and instrumentation, Two-dimensional materials, Electronic properties and materials

## Abstract

Time-resolved momentum microscopy is an emerging technique based on photoelectron spectroscopy for characterizing ultrafast electron dynamics and the out-of-equilibrium electronic structure of materials in the entire Brillouin zone with high efficiency. In this article, we introduce a setup for time-resolved momentum microscopy based on an energy-filtered momentum microscope coupled to a custom-made high-harmonic generation photon source driven by a multi-100 kHz commercial Yb-ultrafast laser that delivers fs pulses in the extreme ultraviolet range. The laser setup includes a nonlinear pulse compression stage employing spectral broadening in a Herriott-type bulk-based multi-pass cell. This element allows flexible tuning of the driving pulse duration, providing a versatile time-resolved momentum microscopy setup featuring two operational modes designed to enhance either the energy or time resolution. We show the capabilities of the system by tracing ultrafast electron dynamics in the conduction band valleys of a bulk crystal of the 2D semiconductor WS_2_. Using uncompressed driving laser pulses, we demonstrate an energy resolution better than (107 ± 2) meV, while compressed pulses lead to a time resolution better than (48.8 ± 17) fs.

**Supplementary Information:**

The online version contains supplementary material available at 10.1038/s41598-025-86660-1.

## Introduction

Angle-resolved photoelectron spectroscopy (ARPES) is a powerful technique for probing the band structure of surfaces, allowing the simultaneous measurement of the kinetic energy and quasi-momentum of photoemitted electrons^1^. To map the electronic band structure in the entire first surface Brillouin zone (BZ), a photon energy exceeding 20 eV is typically required, as the maximum accessible momentum vector depends on the kinetic energy of the photoelectrons^2^. Although laser-based laboratory table-top setups have traditionally been limited either to photon energies below 10 eV through frequency conversion in nonlinear optical crystals^3–5^or low-repetition-rate lasers for efficient high-harmonic generation (HHG)^6–10^, recent advancements in high-average-power and high-repetition-rate laser sources have enabled the development of high-flux ARPES measurements using HHG^11–14^. In addition, pulsed light sources enable the study of nonequilibrium carrier and lattice dynamics on an ultrafast timescale, achieved by employing an additional optical pump pulse with an adjustable relative delay to the probe pulse. Such time-resolved ARPES (tr-ARPES) studies encompass a broad spectrum of materials and phenomena^15,16^, including topological insulators^4,17,18^, high-temperature superconductors^19^, charge density wave insulators^20,21^, and multiparticle excitations, such as excitons^22–24^and orbital d-d transitions^25^.

The introduction of momentum microscopes for photoelectron detection marks another significant step in tr-ARPES experiments. Conventional ARPES analyzers capture a narrow electron momentum cut in the photoelectron distribution and subsequently filter electrons by energy^26^. However, this approach requires varying the azimuthal and polar angles of the sample to map larger momentum space areas, such as the entire first BZ. This limitation can be overcome in photoemission electron microscopes (PEEM) operating in momentum mode, also called momentum microscopes (MM). Here, photoelectrons are accelerated by a high electric field (> 10 kV) towards the objective lens of the microscope, drastically increasing its angular acceptance. When operating in real-space mode instead, MMs can probe the real-space distribution of the photoelectrons. The unique combination of the two operation modes within the same instrument offers the additional advantage of performing position-dependent ARPES scans on small sample sizes, such as exfoliated structures of van der Waals layered materials^27–29^.

One possibility to detect photoelectrons in a MM is to use a hemispherical analyzer, which categorizes the electron kinetic energy by their trajectories through an electrostatic field. Hemispherical analyzers enable usage of continuous-wave light sources with high flux and narrow bandwidth for static experiments but require scanning the kinetic energies individually. A second possibility to discriminate the kinetic energy of the photoelectrons is to equip the MM with a time-of-flight (TOF) detector, which determines the electron kinetic energy by time-of-flight in a drift tube. This allows simultaneous mapping of electron momenta and energies in parallel, significantly reducing the required integration time^11^. In addition, TOF detectors provide a high dynamic range and energy resolution^9^. While being a viable alternative or add-on to MM detectors using a hemispherical analyzer, they require pulsed light sources with appropriate repetition rates, which typically limit the energy resolution of the instrument to the available bandwidth of the laser pulses^26^and the XUV photon energy used^16^.

In general, in photoemission experiments, the acquisition time is primarily dictated by the number of emitted electrons per second. However, in pulsed laser-assisted photoemission, exciting multiple electrons per optical pulse initiates Coulomb interactions between them. This interaction not only significantly shifts the acquired spectrum, but also compromises the energy resolution, leading to the so-called space charge limit^26,30–32^. Overcoming this problem requires extreme ultraviolet (XUV) sources with high repetition rates, preferably in the multi-100 kHz to 1 MHz range, depending on the system to be studied. With respect to the required pulse duration, a compromise needs to be met: although a short pulse duration is required to achieve good time resolution and conversion efficiency to the XUV, the resulting broadband pulses limit the energy resolution of the experiment, which is a well-known compromise to be made in tr-ARPES experiments. HHG without additional pulse compression addresses this limitation, achieving energy resolutions down to a few tens of meV^33–35^, albeit with repetition rates typically below 250 kHz and low conversion efficiency due to well-known pre-ionization effects in HHG. In particular, a light source with switchable energy and time resolution to exploit both advantages has only been reported with photon energies of up to 7.2 eV^36^or without integration into a tr-ARPES setup^14^.

In this context, ultrafast Yb lasers have been recognized several decades ago to provide an ideal platform for generating high-repetition-rate XUV pulses^37^. Coupling these Yb lasers to passive enhancement cavities allows access to XUV sources with repetition rates > 10 MHz and a high usable flux^12,13,38,39^. Another more widespread approach with larger flexibility, particularly at repetition rates in the hundreds of kilohertz targeted here, involves external nonlinear post-compression of the optical pulses generated by high-repetition-rate Yb laser systems to generate XUV photons. To date, this has been the main method of choice for tr-ARPES, and setups with repetition rates up to 1 MHz and pulse durations of the order of 50 fs have been realized^2,11,40^. External nonlinear pulse compression employs self-phase modulation (SPM), induced by the Kerr effect, to spectrally broaden narrow-bandwidth pulses, thus supporting a shorter transform-limited temporal duration. The pulses can subsequently be recompressed in time by eliminating residual chirp from SPM and other dispersion sources. For high pulse energies (millijoule regime), most standard compression setups are gas-filled hollow-core capillaries^41^and filament compressors^42^. However, these methods are difficult to scale in terms of repetition rate and average power without increasing complexity and size, and their overall efficiencies are low, which is highly critical when HHG is targeted at high repetition rates, where the pulse energies available are more moderate.

In this context, recent advancements utilizing Herriott-type multi-pass cells (MPCs) as a free-space alternative for high-average-power compression have revolutionized the high-average-power ultrafast field^43^. The concept behind an MPC is that of a free-space quasi-waveguide, where SPM can be accumulated over successive passes in a compact Herriott cell with a nonlinear medium inside (gas or solid) without beam degradation. These innovative designs achieve remarkable compression ratios and high transmission while simultaneously preserving excellent power efficiency and maintaining pristine spatial beam quality. Moreover, this approach exhibits exceptional versatility by accommodating a wide range of pulse energies, spanning from microjoules to hundreds of millijoules, by adjusting the nonlinear medium employed within the cell^44–47^. However, to the best of our knowledge, such promising pulse compression setups and their wide flexibility have not yet been used for tr-ARPES. In fact, most Herriott-type compression setups have so far not been used for applications and remain in the proof-of-principle for the laser community, except in terahertz generation experiments, such as the one reported in Ref^48^.

In this work, we combine recent advances in external nonlinear pulse compression using Herriott-type cells and in ARPES detectors into a tr-ARPES system. An Yb-ultrafast laser generates 21.6 eV photons via HHG at high repetition rates in the multi-100 kHz regime and is coupled to a MM equipped with a hemispherical analyzer. The employed compact nonlinear compression scheme allows for pulse compression below 50 fs to enhance the time resolution but can also be bypassed to achieve an energy resolution better than 107 meV. In addition, we coupled a monochromatized helium discharge light source to the hemispherical analyzer for high-flux characterization of the ground state. To demonstrate the performance of this flexible setup for tr-ARPES, we tracked the ultrafast dynamics of conduction-band electrons in a bulk crystal of the well-known 2D quantum material WS_2_.

## Experimental setup

### Nonlinear pulse compression

We begin by describing the central part of our experiment, that is, the custom-built setup for nonlinear pulse compression. The optical pulse duration is a crucial parameter in tr-ARPES experiments since it defines the time resolution but also heavily influences the HHG process via optical peak power. Short pulses (typically < 100 fs) are known to improve the HHG yield but diminish the energy resolution of the tr-ARPES measurements. To obtain such short pulses from Yb laser systems that typically produce > 200 fs pulses, external nonlinear pulse compression is required.

**Fig. 1:**
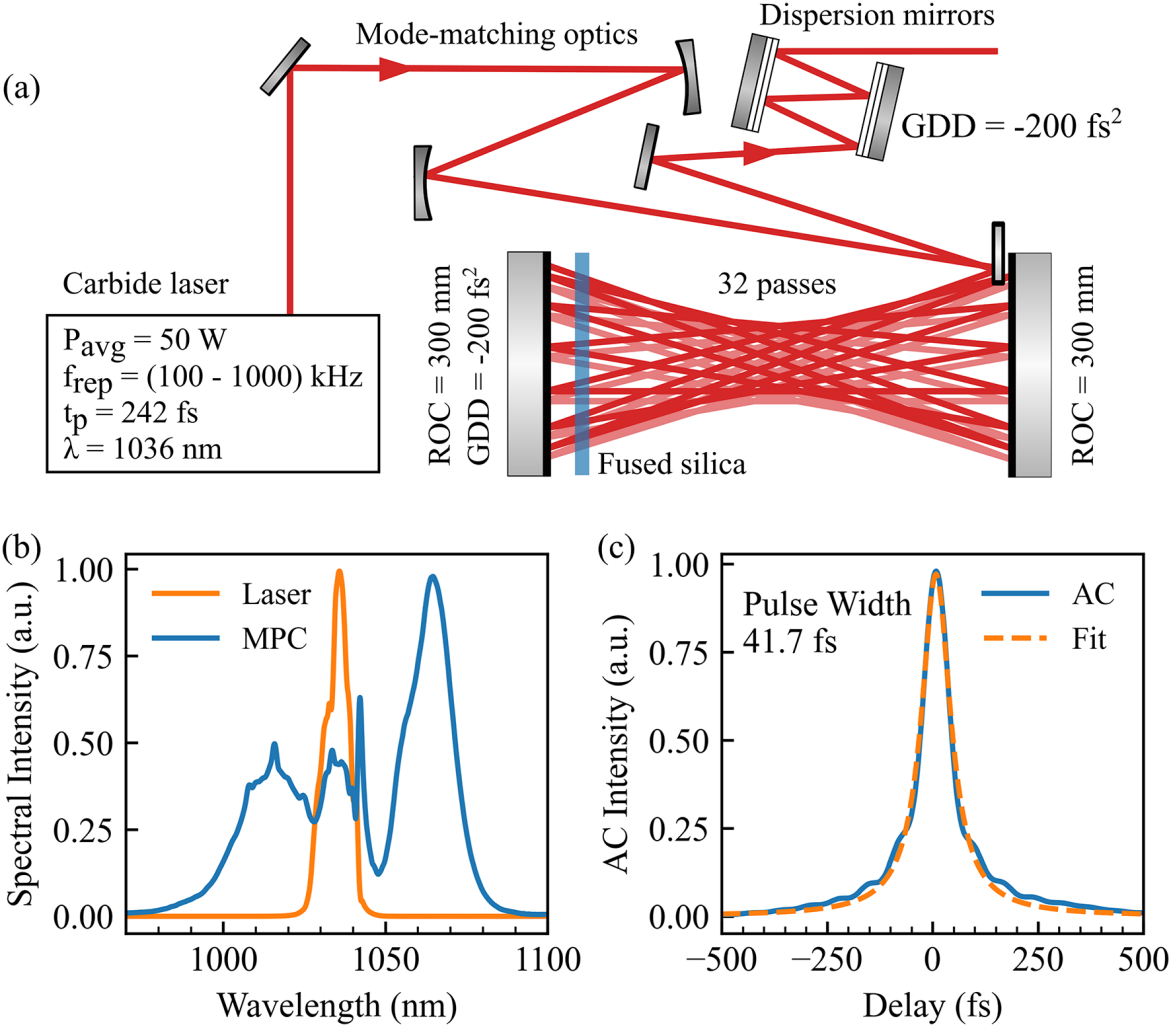
The nonlinear pulse compression setup. The output pulses of the commercial fiber laser “Carbide” are compressed with a bulk Herriot-type multi-pass cell and dispersion correcting mirrors as displayed in (**a**). (**b**) compares the laser output to the accumulated spectral broadening after the MPC, which allows for pulse durations below 50 fs as depicted in an autocorrelation measurement and fitted Lorentzian function in (**c**).

The optical layout of our hybrid bulk-air MPC setup is depicted in Fig. 1a, which utilizes the output of a commercial 50 W ultrafast laser (Carbide, Light Conversion) with a variable repetition rate from 100 kHz to 1 MHz, a central wavelength of 1036 nm, and a temporal pulse duration of 242 fs. For compression of the spectrally broadened laser pulses, the repetition rate is set to 600 kHz, and the pulses with 83 µJ pulse energy are first steered into a Herriot-type MPC that comprises two highly reflective (HR) concave mirrors with a radius of curvature (ROC) of 300 mm and an anti-reflection coated fused silica (FS, d = 6.35 mm) in between. For a total of 32 passes through the nonlinear medium (16 roundtrips), the optical pulse accumulates spectral broadening via self-phase modulation in the FS. Mode-matching optics before the MPC provide a uniform caustic between both mirrors to achieve a significant spectral broadening that supports 50 fs and ensures good beam mode quality. Figure 1b displays the spectrum before and after the MPC, revealing significant spectral broadening from 22.2 nm to 68.5 nm at −20 dB. However, pulse propagation through the FS plate accumulates a group dispersion delay (GDD) of the optical pulse, which is partly compensated by the coating of one MPC mirror with GDD = −200 fs^2^.

The beam then undergoes 12 reflections on a dispersive mirror pair with − 200 fs² per reflection and an overall GDD of −2400 fs², compressing the pulse to 41.7 fs as presented in Fig. 1c. The figure shows the measured autocorrelation next to a Lorentzian fit. In combination with an overall high transmission of 86% giving a pulse energy of 71.7 µJ, the short pulse duration allows for a peak power > 1.3 GW optimal for HHG. This peak power is the result of a simulation of the MPC performance as described in detail in section S2. In addition, the high-quality output mode of the laser is preserved with $\:{M}_{u}^{2}=1.17$ and $\:{M}_{v}^{2}=1.23$ after compression (compare section S1), which allows for tight focusing to further boost the harmonic generation efficiency.

### Coherent extreme ultraviolet photon generation

**Fig. 2:**
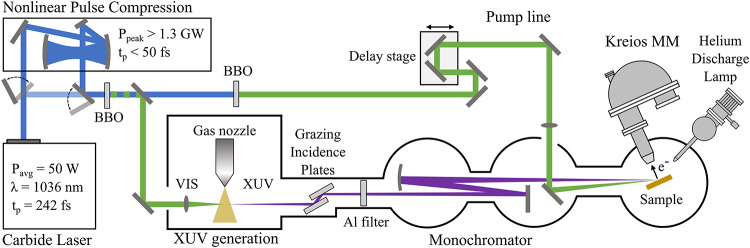
Schematic view of the time-resolved ARPES setup. A 50 W Yb-ultrafast laser with optional compression is split into a pump line to excite non-equilibrium states and a probe line to generate high harmonics. The high harmonics are monochromized via grazing incidence plates and multilayer optics before impinging on the sample surface. Photoemitted electrons are measured using a Kreios MM equipped with a hemispherical analyzer for kinetic energy discrimination. The MM allows imaging of the electron distribution in both real and momentum space.

Figure 2 shows a schematic of the photoemission setup, which is based on a Carbide laser, with a variable repetition rate from 100 kHz to 1 MHz. The design offers two configuration options, allowing flexibility for experiments focused on either temporal or energy resolution. The laser output is either directed into the nonlinear pulse compressor described earlier, or users can bypass the compression module, preserving the narrow spectral bandwidth of the laser for higher energy resolution. We chose to conduct the experiments at 600 kHz to optimize the high harmonic yield and facilitate the future integration of an optical parametric amplifier (OPA). However, the use of an MPC for pulse compression allows for adaptation to different repetition rates if needed.

Following the optional compressor stage, the infrared pulses undergo frequency doubling to a wavelength of 515 nm using a 0.5 mm thick beta barium borate (BBO) crystal. The conversion efficiency is 27.9% for compressed and 59.0% for uncompressed pulses, yielding a pulse energy of 20 µJ and 49.1 µJ, respectively. A dichroic mirror is then employed to separate the original infrared light from the newly generated green light. High stability of the optical setup is particularly crucial for time-resolved scans of the photoemission spectrum. While static spectra can be acquired within a few hours (e.g., with an integration time of 72 s per energy point in Fig. 4), transient signals require significantly longer integration times, extending up to several days (e.g., 500 s per energy and delay step in Fig. 6). We use active beam stabilization (Aligna, TEM Messtechnik) before dividing the pump and probe pulses to mitigate both short-term instabilities and thermal beam walk-off. As a result, thermal beam drift is small, with a count rate decrease of 30% after 4 h.

**Fig. 3:**
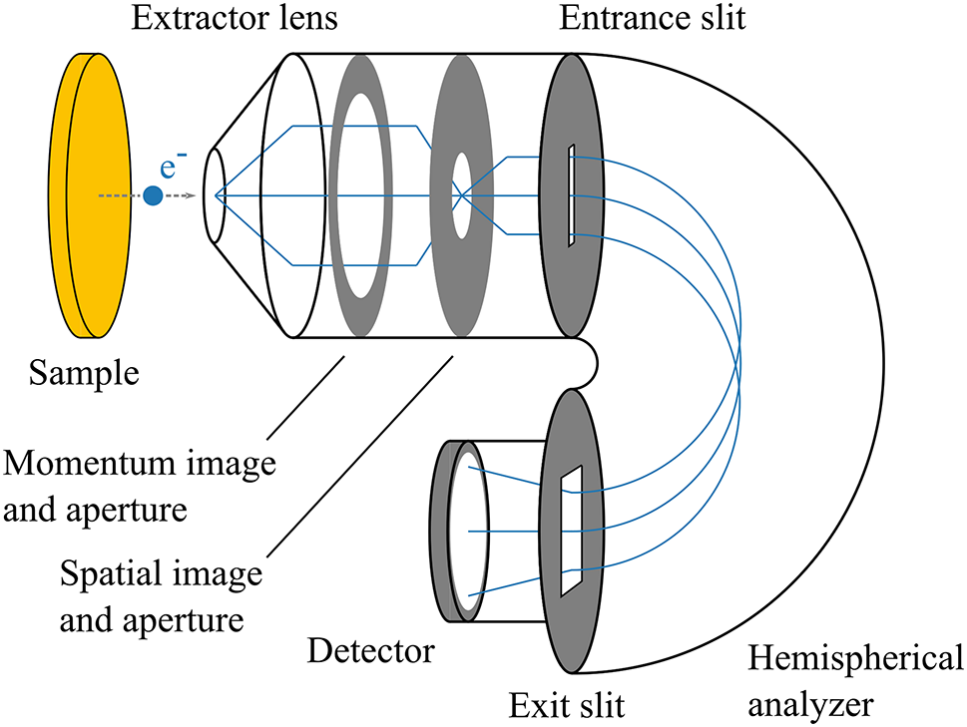
Schematic diagram of the MM in momentum mode.

**Fig. 4:**
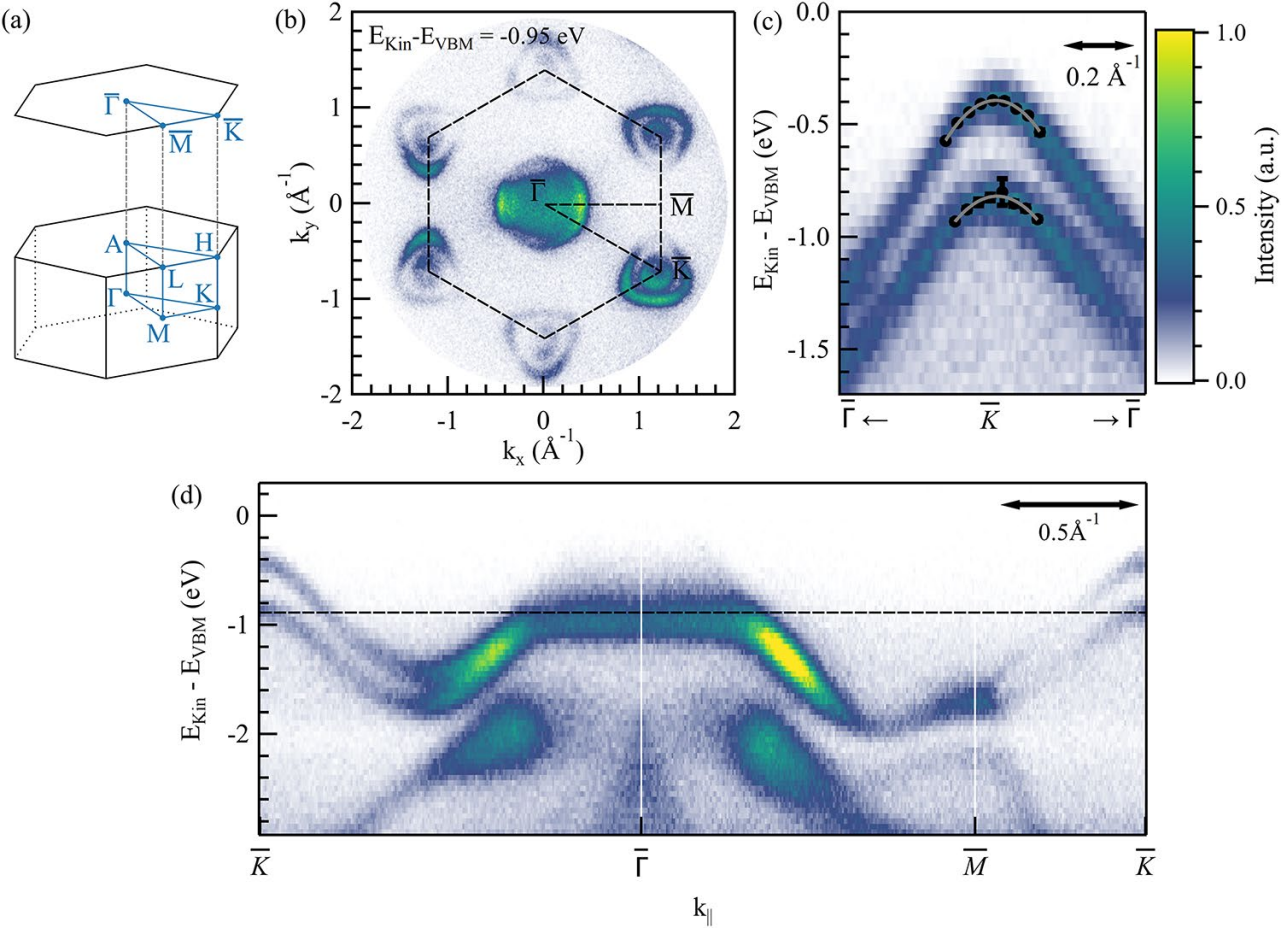
Static ARPES spectrum of bulk WS_2_ measured with uncompressed XUV pulses at room temperature. (**a**) Bulk and (0001)-surface BZ with high symmetry points for WS_2_. (**b**) Momentum map recorded at 0.95 eV below the valence band maximum (VBM). The black dashed hexagon marks the border of the first BZ, dashed lines mark additionally two paths along high symmetry points along which we display the ARPES intensity in (**d**). (**c**) Zoom in around the $\:\stackrel{-}{\text{K}}$ point. The black dots mark the center of fitted Gaussian distributions to vertical cuts, used to extract the effective mass of the bands.

The non-converted infrared pulses are harnessed as a pump pulse, while the green light produced by the second harmonic generation is focused using a 20 cm focal length lens into an Argon (Ar) gas jet, initiating the production of XUV photons through HHG in a XUV generation chamber supplied by Active Fiber Systems GmbH. The Ar gas jet emanates from a nozzle with a diameter of 200 μm and is maintained at a backing pressure of approximately 1 bar. Post HHG, a pair of grazing incidence plates filters the driving 515 nm light into a beam dump, and an aluminum filter with 0.2 μm thickness blocks residual photons of wavelengths higher than 62 nm (which equates to 20 eV photon energy). To monochromatize the XUV light, two bandpass-coated multilayer mirrors are utilized to selectively transmit the 9th harmonic at 21.6 eV, effectively filtering out adjacent harmonics by at least a factor of 10. The second mirror in the monochromator, with a ROC of 2 m, focuses the linearly *p*-polarized XUV light onto the sample to a beam size of 19.5 μm x 31.7 μm (see section S4 for details). Although direct measurement of the photon flux at the sample is not feasible, we consistently reach the space charge limit (< 1 electron/pulse) across all tested substrates (HOPG, Au(111), and WS_2_), ensuring optimal flux for trARPES experiments. To reliably tune the XUV flux to the onset of the space charge limit, we monitor the photoemission spectrum while systematically reducing the Ar gas pressure and increasing the distance between the Ar nozzle and the laser focus to decrease the photon flux artificially. As photoelectron spectroscopy requires ultrahigh vacuum (UHV) conditions, we reduce the base pressure from $\:1\times\:{10}^{-2}$ mbar in the HHG chamber to $\:1\times\:{10}^{-10}$ mbar in the analysis chamber through differential pumping.

To perform tr-ARPES, we implemented a pump-probe scheme, where a pump pulse induces a non-equilibrium state within the system, and a subsequent XUV probe pulse captures the system dynamics at specific time intervals (Δt) in a stroboscopic manner. In our setup, the nonconverted infrared light remaining from the second-harmonic generation process serves as the pump. The pump beamline includes a motorized delay stage, enabling precise adjustment of the temporal gap between the pump and probe pulses to explore dynamic processes over time. The pump beam is focused onto the sample by a 2 m ROC lens, achieving a beam diameter of approximately 300 μm on the sample. An in-coupling mirror in the monochromator enables nearly collinear propagation of the pump and probe beams, impinging on the sample at an incidence angle of 22°. Unless otherwise specified, the pump beam is *p*-polarized. Additionally, the pump photon energy, originally at 1.2 eV, can be frequency-doubled using a 0.5 mm thick BBO crystal. The experimental configuration is designed to accommodate an OPA, offering the capability to precisely fine-tune the pump energy. This level of customization and control is crucial for probing a wide range of material responses under various excitation conditions.

### Momentum microscope

The photoemission end station comprises two UHV chambers, both with a base pressure of $\:1\times\:{10}^{-10}$ mbar. One holds the MM along with a sample stage mounted on a hexapod with six degrees of freedom, which can be cooled by either liquid helium or nitrogen through a flow cryostat. Attached to this analysis chamber is a separate preparation chamber (not shown in Fig. 2) with standard equipment for sample cleaning, preparation, and characterization by low energy electron diffraction (LEED) and Auger electron spectroscopy (AES).

We implemented a commercial MM equipped with a hemispherical analyzer (Kreios 150, Specs GmbH). This instrument was introduced by some of the authors in Reference^4^ and is displayed in Fig. 3. In addition, in S5 we benchmarked this instrument by performing additional static measurements on an Au(111) surface at 6.5 K with 6 eV photons and obtained 49 meV for the energy resolution and 0.005 Å^−1^ for the momentum resolution. In the Kreios MM, an extractor lens with a 10 kV potential difference relative to the sample deflects the photoemitted electrons into the optical column. Specifically designed electrostatic lenses generate two image planes comprising the real and momentum-space distributions. The electrons are subsequently filtered by energy in the hemispherical analyzer with an adjustable entrance slit size and pass energy for an energy resolution down to 22 meV. Finally, the energy-filtered electrons are projected onto a 2D detector. This measuring scheme allows for specified lateral and angular fields of view of ± 3 Å^−1^ and 200 μm with resolutions of 36 nm and 0.0050 Å^−1^, respectively. By placing apertures in the focal planes, a small sample surface area (down to 2 μm in diameter) can be selected for the acquisition of photoemission spectra, allowing the study of samples with small lateral sizes, for example, exfoliated structures of 2D-layered materials. In contrast to time-of-flight detectors, in which the kinetic energy of the photoelectrons is determined by the flight duration in a drift tube, hemispherical analyzers do not require a pulsed light source. In addition to the laser system described above, a monochromatized helium discharge lamp (UVS 300, Specs GmbH) with a linear *p*-polarized output can thus be coupled to the microscope. The continuous wave emission and adjustable monochromator allow high-resolution static photoemission spectroscopy without space charge effects.

**Fig. 5:**
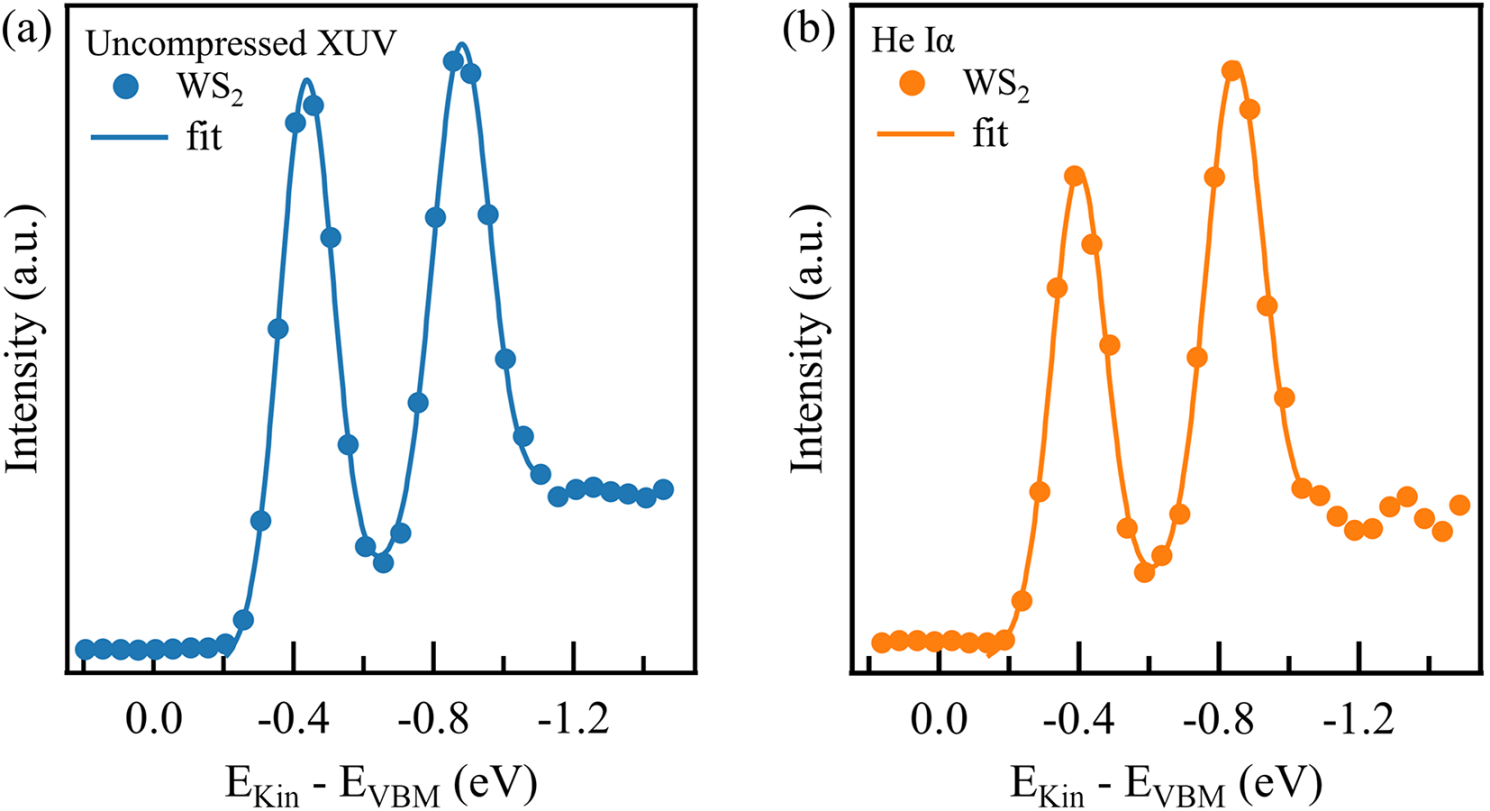
The spin-split valence band of WS_2_ at the $\:\stackrel{-}{\text{K}}$ point. The measurements were carried out using uncompressed XUV pulses in (**a**) and the helium discharge lamp in (**b**). Both curves were fitted with two Gaussian distributions and a linear background that give the same result for band widths and band splitting energy.

The resolution of the microscope in terms of energy, momentum, and lateral resolution is contingent upon the configuration of the lens system, the size of the entrance slit, and the pass energy of the hemispherical analyzer. Nevertheless, achieving the highest resolution entails employing high magnifications and narrow entrance slits, which significantly reduce the photoelectron count rate, necessitating high repetition rates, such as the 600 kHz rate utilized in this study. In the following section, we showcase the performance of the integrated XUV light source and MM using a bulk WS_2_ sample system. With the uncompressed XUV pulses, the mode for high energy resolution, we achieve an energy resolution of 107 meV; the compressed XUV pulses, generated for high temporal resolution, deliver a time resolution of 49 fs.

## Showcasing the setup with bulk WS2

To demonstrate the capabilities of our setup for tr-ARPES, we performed benchmark experiments on a bulk WS_2_ crystal. WS_2 _is a member of the heavily studied family of transition metal dichalcogenides (TMDs), which are 2D semiconductors with large spin-orbit coupling and large exciton binding energies due to reduced electronic screening that governs the optical response^49–51^. Our selection of this material was driven by two main factors: the desire to evaluate the performance of our system on a 2D quantum material, thereby utilizing the lateral resolution of our instrument, and the availability of existing tr-ARPES data on WS_2_, which allows for direct comparison with our results.

### Static momentum microscopy using uncompressed XUV pulses

Figure 4 displays the static ARPES spectra measured at room temperature with the uncompressed XUV pulses for enhanced energy resolution (pass energy of 50 eV, entrance slit of 0.5 × 1.0 mm). The complete first BZ, close to the valence band maximum (VBM) energy, is within the experimental field of view with the chosen photon energy of the XUV pulses and microscope settings (see Fig. 4a and b). The MM acquires isoenergetic contours as the one shown in Fig. 4b, corresponding to 0.95 eV below the VBM. Such “momentum maps” are recorded in one shot without the need to scan the polar and azimuthal angles. The black dashed lines mark the edge of the first BZ and pathways between high-symmetry points. A cut along these indicated high-symmetry lines is shown in Fig. 4d. At the VBM, the acquired intensity at $\:\stackrel{-}{{\Gamma\:}}$diminishes, contradictory to previous photoemission experiments at higher photon energies^49,52^and theoretical calculations^49,53,54^. This behavior has already been reported in other van der Waals systems at photon energies comparable to those used here, and is likely to originate in a vanishing transition matrix element^55,29^. Overall, the momentum map in Fig. 4b contains approximately $\:6.6\times\:{10}^{5}$ counts, corresponding to a detection rate of about 9200 counts per second, given the integration time of 72 s per energy step.

The high spin-orbit coupling strength in WS_2_ results in a splitting of the valence band at the $\:\stackrel{-}{\text{K}}$ point, which is analyzed in Fig. 4c. To determine the effective mass of the depicted bands, we fitted vertical cuts along the band structure with Gaussian distributions and took their center as the energetic position of the band. By fitting those points with a parabola, we find an effective mass of $\:\left(-0.39\pm\:0.01\right){\text{m}}_{0}$ and $\:\left(-0.43\pm\:0.05\right){\text{m}}_{0}$ in the upper and lower band, respectively ($\:{\text{m}}_{0}$ is the electron mass). This fit is displayed as a grey line in Fig. 4c and agrees with previous findings of $\:\left(-0.35\pm\:0.02\right){\text{m}}_{0}$ and $\:\left(-0.43\pm\:0.07\right){\text{m}}_{0}$ by I. Tanabe et al.^52^. Next, we established an upper limit for the energy resolution by comparing the spectra obtained using uncompressed XUV pulses or the helium discharge lamp as a light source, respectively. Figure 5 displays the intensity profiles of the two sub-bands at the $\:\stackrel{-}{\text{K}}$ point, which were extracted by summing the intensities of all six $\:\stackrel{-}{\text{K}}$ points within a radius of 0.07 Å^−1^ each. Both curves were fitted with two Gaussian distributions and a linear background to determine the bandwidths and their splitting. The uncompressed XUV measurement yielded bandwidths of (107 ± 2) meV for the upper band and (113 ± 3) meV for the lower band, along with a band splitting of 442 meV. The He Iα measurement yields bandwidths of (108 ± 5) meV and (114 ± 6) meV for the upper and lower bands, respectively, with a band splitting of 446 meV. Both sets of results are closely aligned, indicating that the measured widths are not related to the light source used, but rather to the sample quality or to the MM settings, such as the pass energy and the entrance slit width. We thus conclude that the reported values can be regarded as an upper limit for the energy resolution achievable with uncompressed XUV pulses. In contrast, the energy resolution of the compressed XUV pulse determined in section S3 is 233 meV.

**Fig. 6:**
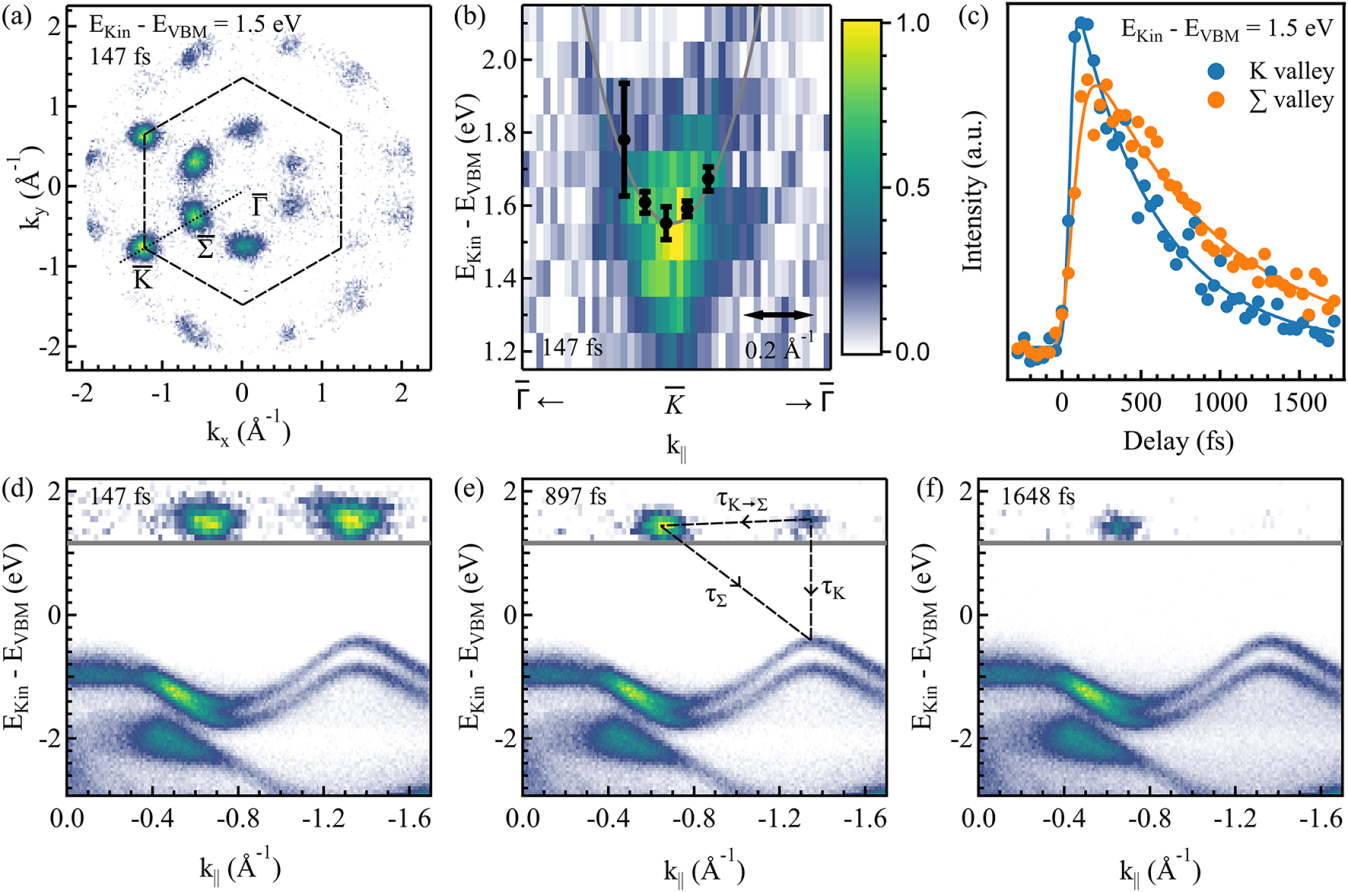
Transient occupation and dynamic of different conduction band valleys. (**a**) Momentum map of the conduction band at 1.5 eV above the VBM, recorded 147 fs after optical excitation. The data is subtracted by the equilibrium state at negative delays. Black dashed lines mark the edge of the first BZ and a dotted line marks a cut along high-symmetry points, which is presented in the top half of (**d**)–(**f**) for different time delays. The bottom spectra present photoemission intensity measured in the static ARPES experiments with the uncompressed XUV pulses along the same path in momentum space. (**b**) Dispersion of the $\:\stackrel{-}{\text{K}}$ valley with fitted Gaussian distributions as black dots together with the dispersion calculated by R. Oliva et al.^60^ (solid gray line). (**c**) Temporal evolution of the population of the $\:\stackrel{-}{\text{K}}$ and $\:\stackrel{-}{{\Sigma\:}}\:$valleys, plotted with arbitrary intensity units.

### Time-resolved momentum microscopy with compressed XUV pulses

We now discuss tr-ARPES experiments performed with *p*-polarized 2.4 eV pump pulses at a fluence of 471.57 µJ/cm² to photoexcite electrons in the conduction band of WS_2_ and study their ultrafast relaxation dynamics. Bulk WS_2_ hosts a conduction band with local minima at different points in the BZ, for example, the $\:\stackrel{-}{\text{K}}$ and the $\:\stackrel{-}{{\Sigma\:}}$points. While excited carrier dynamics in these valleys were subject to optical studies, only ARPES measurements were able to directly image transient dynamics for different valleys individually^50,51,56^. With the high pump fluence used in our experiments, we excite approximately $\:7.4\times\:{10}^{13}\:{\text{c}\text{m}}^{-2}$free carriers. In this regime, exciton formation is inhibited and band renormalization has been observed^57,58^. In the following section, we use compressed pulses to enhance the temporal resolution of the experiment.

Figure 6a shows a transient momentum map measured at 1.5 eV above the VBM with the compressed XUV pulses (pass energy 50 eV, entrance slit 1.0 × 1.8 mm). The map was recorded at a time delay of 147 fs after optical excitation, and the background was subtracted by the corresponding momentum map recorded at negative delays (unperturbed state). This subtraction eliminates the intensity contributions above the VBM stemming from the leakage of higher harmonics. After subtraction, the map in Fig. 6a contains approximately $\:3.8\times\:{10}^{5}$ counts, corresponding to a detection rate of about 760 counts per second, given the integration time of 500 s per energy step. We observe a strong signal in the $\:\stackrel{-}{\text{K}}$ and $\:\stackrel{-}{{\Sigma\:}}$ valleys, whose position is also given in the figure, with a strong gradient from the left to the right of the image, which stems from the photon incident direction and angle. A dotted black line marks a cut along high-symmetry direction in the first BZ. The photoemission intensity along this line, stemming from the states above the VBM, is displayed in Fig. 6d–f for different delays of 147 fs, 897 fs, and 1648 fs. Here, the photoemission intensity measured in the static ARPES experiments with uncompressed XUV pulses (compare Fig. 4) below the VBM is added to provide a complete picture of both the ground and excited states. Visual inspection of the figures already provides evidence of the longer lifetime of the electrons occupying the $\:\stackrel{-}{{\Sigma\:}}$ valley compared to those in the $\:\stackrel{-}{\text{K}}$ valley, which is due to the different scattering mechanisms active in the two valleys. In fact, while hot electrons in the $\:\stackrel{-}{\text{K}}$ valley can directly recombine with holes at the $\:\stackrel{-}{\text{K}}$ point, $\:\stackrel{-}{{\Sigma\:}}$ valley electrons only experience indirect recombination with holes at the $\:\stackrel{-}{{\Gamma\:}}$ or $\:\stackrel{-}{\text{K}}$ point, as observed in previous studies^50,56,59^.

**Fig. 7:**
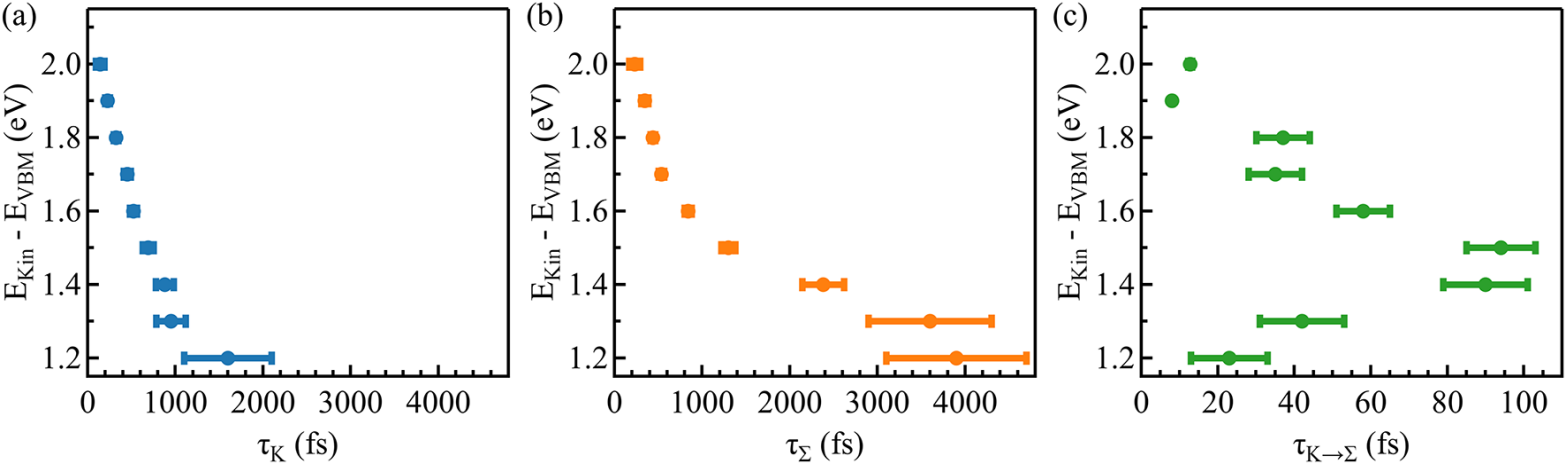
Energy-dependent valley scattering times. (**a**) In the $\:\stackrel{-}{\text{K}}$ valley, and (**b**) in the $\:\stackrel{-}{{\Sigma\:}}$ valley, the relaxation time increases monotonically towards the conduction band minimum. (**c**) The intervalley scattering exhibits decreasing relaxation times below 1.5 eV, likely due to scattering channels not included in the model.

To quantify this behavior, we plotted in Fig. 6c the transient population of the hot electrons in the two valleys at 1.5 eV above the VBM, calculated by integrating the photoemission intensity of the $\:\stackrel{-}{\text{K}}$ and $\:\stackrel{-}{{\Sigma\:}}$points within a region of 0.5 Å^−1^ each. As depicted by the arrows in Fig. 6e, we modeled the curves by assuming that the population of the $\:\stackrel{-}{\text{K}}$ valley can either recombine (decay time $\:{\tau\:}_{\text{K}}$) or scatter into the $\:\stackrel{-}{{\Sigma\:}}$ valley (decay time $\:{\tau\:}_{\text{K}\to\:{\Sigma\:}}$). Both decay times, $\:{\tau\:}_{\text{K}}$ and $\:{\tau\:}_{\text{K}\to\:{\Sigma\:}}$ are assumed to be energy-dependent (details presented in section S6). For the population of the $\:\stackrel{-}{{\Sigma\:}}$ valley, we used a single energy-dependent decay time $\:{\tau\:}_{{\Sigma\:}}\:$. The extracted energy-dependent decay times resulting from electron-phonon and intravalley electron-electron scattering^56,61^ are shown in Fig. 7. At an exemplary energy of 1.5 eV (see Fig. 6c), we found relaxation rates of $\:{\tau\:}_{\text{K}}=\left(690\pm\:60\right)$ fs for the $\:\stackrel{-}{\text{K}}$ valley, $\:{\tau\:}_{\text{K}\to\:{\Sigma\:}}=\left(94\:\pm\:\:9\right)$ fs for intervalley scattering, and $\:{\tau\:}_{{\Sigma\:}}=\left(1300\pm\:80\right)$ fs for the $\:\stackrel{-}{{\Sigma\:}}$ valley. For both the$\:\stackrel{-}{\text{K}}$ and $\:\stackrel{-}{{\Sigma\:}}$ valley, the relaxation rate monotonously increases towards the valley minimum, whereas the intervalley scattering rate $\:{\tau\:}_{{\Sigma\:}\to\:K}$ has non-monotonous behavior, with a maximum at 1.5 eV.

Since we modeled the electron distribution of the $\:\stackrel{-}{\text{K}}$ valley to stem exclusively from direct optical excitation (an instantaneous event), we could determine the time resolution of the experiment by fitting the increase in the transient spectral intensity. We fitted a convolution of an exponential decay multiplied with the Heaviside function and a Gaussian distribution, comprising the cross-correlation of the visible pump and XUV probe beam and the sample response time. For the curve displayed in Fig. 6c, the Gaussian full width at half maximum measures (48.8 ± 17) fs, providing an upper limit of the overall temporal resolution. The temporal resolution of the tr-ARPES experiments with the uncompressed XUV pulse was determined in section S3 to (181 ± 5) fs, demonstrating a remarkable improvement in the temporal resolution obtained with the compression stage.

Finally, we tracked the band dispersion of the $\:\stackrel{-}{\text{K}}$ valley in Fig. 6b by fitting a Gaussian distribution at different momenta, similar to the valence band maxima before (compare Fig. 4c). The fitted center positions are plotted as black dots and prove excellent agreement with the previously calculated effective mass of $\:{m}_{\text{c}}=0.36\:{m}_{0}$ by Oliva et al.^60^ (grey line).

With a measured temporal resolution of 48.8 fs and an energy resolution of 233 meV (as described in section S3), the time-bandwidth product computes to 11,370 $\:\text{m}\text{e}\text{V}\times\:\text{f}\text{s}$ and exceeds the Fourier limit of 1825 $\:\text{m}\text{e}\text{V}\times\:\text{f}\text{s}$^16^. While achieving the Fourier limit is challenging in practice, a comparable trARPES system by Puppin et al. demonstrated a temporal resolution of 40 fs and an energy resolution of 120 meV^62^, facilitated by fine-tuning the time-bandwidth product using an HHG-driven OPCPA system. The deviation in our system could arise from several reasons: First, the bandpass coating of the XUV monochromator might be too broad or misalignment could induce slight geometric dispersion. In contrast to grating monochromators, where the exit slit size and grating groove density tune the transmitted bandwidth^9,40^, multilayer mirrors have a fixed setting and can only be replaced by a set with adapted coating. Second, misalignment or spatial inhomogeneities in the beam profile might increase the pulse duration. Finally, pump pulses longer than the probe XUV pulses compromise the time-resolution in our experiments. Here, additional compression stages could be implemented for the pump to better match the XUV pulse duration. Fine-tuning the monochromator bandpass setting to balance photon flux and energy resolution is also feasible, particularly if the generated harmonics are monitored using an XUV spectrometer. Despite the current limitations, the demonstrated tunability of our setup is valuable for experiments requiring a trade-off between energy and time resolution. For instance, while the hot-electron dynamics presented here were studied with the time-optimized configuration, further investigations could use an energy-optimized configuration to precisely determine intravalley dynamics or relative energetic positions of the respective valleys.

## Conclusion

In summary, we introduced a versatile momentum microscopy setup for tr-ARPES featuring two operational modes designed to enhance both energy and time resolution. By leveraging a high-power laser, we achieved the generation of XUV photon energies ≥ 20 eV at high repetition rates within the multi-100 kHz regime after optional nonlinear post-compression. The resulting photoelectrons were imaged using a MM equipped with a hemispherical analyzer, which grants additional static characterization with a gas discharge lamp. We characterized the setup by effectively tracking the electron dynamics and relaxation across the conduction band valleys of a bulk crystal of the 2D semiconductor WS_2_. The setup also allows for seamless integration of an OPA stage in the future to resonantly excite quasiparticle resonances, thereby further extending its capabilities for probing ultrafast coherent quasiparticle dynamics in quantum materials.

## Electronic supplementary material

Below is the link to the electronic supplementary material.

Supplementary Material 1

## Data Availability

The data supporting the findings of this study are available from the corresponding author, MC, upon reasonable request.
