# Supplementary material for: Time-resolved momentum microscopy with fs-XUV photons at high repetition rates with flexible energy and time resolution
Source: Sci Rep. 2025 Jan 29;15:3611. doi: 10.1038/s41598-025-86660-1 (PMC11775307; doi:10.1038/s41598-025-86660-1)
Supplement: Supplementary file 1 — Supplementary Material 1 [file 41598_2025_86660_MOESM1_ESM.pdf]

Time-resolved momentum microscopy with fs-XUV photons at high repetition rates with flexible energy and time resolution: Supplemental Document

Karl Jakob Schiller[1], Lasse Sternemann[1], Matija Stupar[1], Alan Omar[2], Martin Hoffmann[2], Jonah Elias Nitschke[1], Valentin Mischke[1], David Maximilian Janas[1], Stefano Ponzoni[1,3], Giovanni Zamborlini[1,4], Clara Jody Saraceno[2] and Mirko Cinchetti[1,*]

[1]*Department of Physics, TU Dortmund University, Otto-Hahn-Straße 4, 44227 Dortmund, Germany*
[2]*Photonics and Ultrafast Laser Science, Ruhr University Bochum, Universitätsstrasse 150, 44801 Bochum, Germany*
[3]*Laboratoire des Solides Irradiés, CEA/DRF/lRAMIS, Ecole Polytechnique, CNRS, Institut Polytechnique de Paris, F-91128 Palaiseau, France*
[4]*Institute of Physics, Karl-Franzens-Universität Graz, Universitätsplatz 5, 8010 Graz, Austria*
**mirko.cinchetti@tu-dortmund.de*

S1 Optical mode before and after nonlinear pulse compression

The quality of the beam plays a crucial role in determining its focusability and is instrumental in achieving optimal high harmonic generation (HHG) performance. Figure S1 displays the measured beam caustic before and after passing through the multi-pass cell (MPC). The curve is modeled by the equation

$$w^2(z) = w_0^2 \left[1 + \left(\frac{M^2 \lambda (z - z_0)}{\pi w_0^2} \right)^2 \right]^2 , \qquad (S1)$$

where w_0 represents the minimal beam radius, λ denotes the central wavelength, and z_0 indicates the focus location. Through fitting, the laser output yields an M^2 value of (1.11×1.21), while the MPC output yields (1.17×1.23). The marginal difference between these beam qualities underscores the remarkable preservation of the optical mode achieved through the MPC. However, the M^2 parameter alone does not conclusively demonstrate a clean optical mode; thus, the performance of the MPC is ultimately evaluated by achieving a high HHG yield.

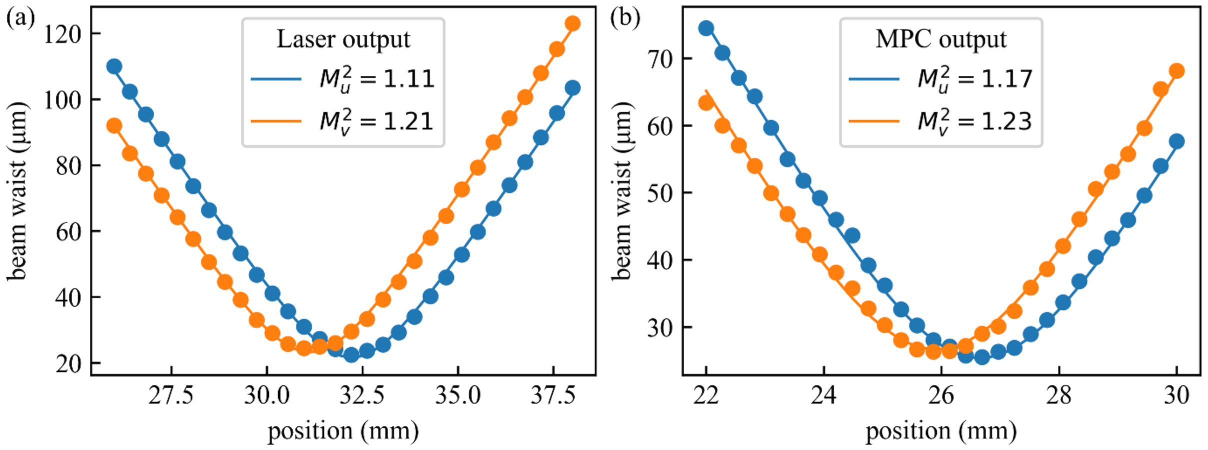

Figure S1. Beam quality factor measurement (M^2) of the laser output a) and the MPC output b). Both measurements show two perpendicular axes u and v, which are fitted with a Gaussian caustic to obtain M^2 parameters.

S2 Simulation of the multi-pass cell performance

We employed a numerical model to design a bulk-based MPC using the available 300-mm concave mirrors in our labs at the time of the experiment. The model involved sweeping the thickness and the position of an anti-reflection coated fused silica (FS) plate across the MPC and adding a group delay dispersion profile to the mirrors to compensate for material dispersion of the FS. Our goal was to achieve spectral broadening that supports sub-50 fs pulse durations while preserving spatial beam quality and maintaining a compact setup to minimize sources of instability for HHG. The model solves the nonlinear Schrödinger equation in three dimensions (x, y, t) using the split-step Fourier method and considers linear effects such as diffraction, dispersion, and loss, as well as nonlinear effects such as the optical Kerr effect, self-steepening, and instantaneous and delayed Raman response.

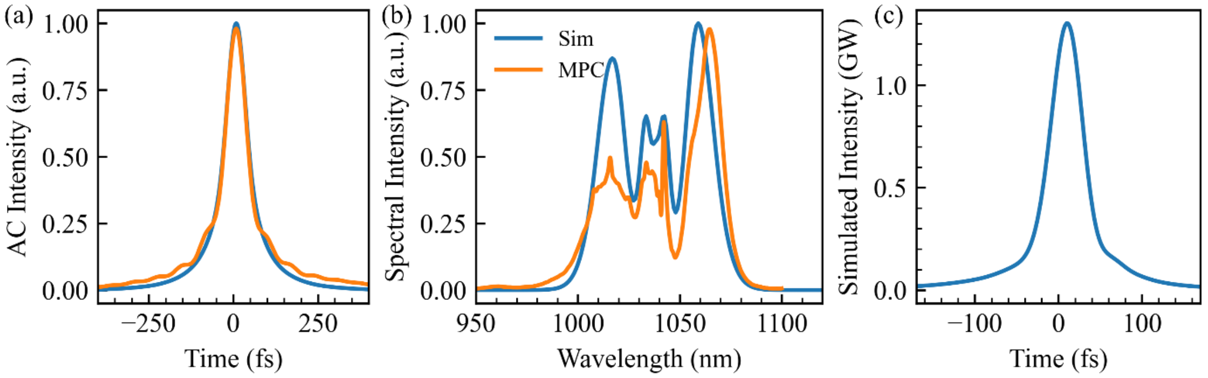

Figure S2. Simulated multi-pass cell compression. Figures a) and b) compare the simulation with the measured autocorrelation and spectrum, respectively. c) Calculated pulse intensity yielding a peak power > 1.3 GW.

Figure S2 displays the calculated pulse characteristics after the nonlinear compression. A direct comparison of the autocorrelation trace in Figure S2a and the spectrum in Figure S2b reveals excellent agreement of the simulation with the measurements. From this conformity, we estimate the output peak

power to be 1.3 GW based on the simulation results (compare Figure S2c). To quantify the spatio-spectral homogeneity of this design, we assess the spectral homogeneity of the simulated compressed beam by calculating the V-parameter [1]. Figure S3a shows the reconstructed spectral distributions along the x-axis. Figure S3b shows the V-parameter (dashed red), with the intensity-weighted average V-parameter being >99 % within a (1/e^2) beam area. These results indicate that this design does not lead to strong spatio-spectral coupling.

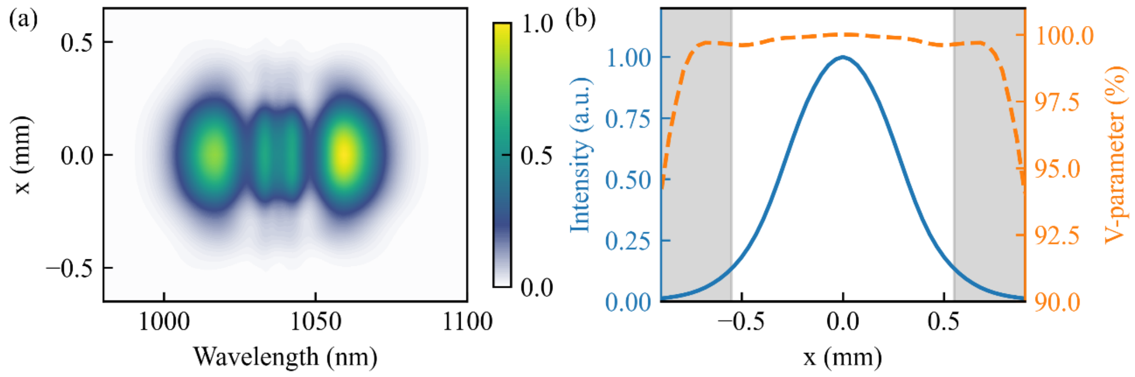

Figure S3. Spatio-spectral homogeneity calculation of the compressed pulse along the x-axis. a) Reconstructed spectral distributions of the simulated compressed pulses along the x-axis. b) Calculated V-parameter, with the solid blue line on the right representing the normalized intensity of the spatial profile. The red dashed line indicates the corresponding spatio-spectral homogeneity value (V-parameter), and the gray boxes denote the beam area within a beam diameter (1/e2).

S3 Comparison of energy- and time-resolution using the compressed and uncompressed XUV pulses

The nonlinear pulse compression of the probe pulses utilizes spectral broadening techniques to allow shorter pulse durations, which in turn have an effect on the energy resolution of ARPES experiments. In the following, we compare both parameters and thus the impact of the compression stage.

The energy and time resolution of the XUV photons at 21.6 eV are displayed in Figure S4. The data in Figure S4a and b was obtained with a 0.2×0.8 mm entrance slit at 50 eV pass energy and shows the angle-integrated photoemission intensity at the Fermi edge of an Au(111) bulk crystal measured at 80 K. The curves are fitted with a Fermi-Dirac distribution for T=80 K convoluted with a Gaussian distribution, to extract the energy resolution. For the measurement performed with the He Iα (Figure S4a), we obtain an energy resolution of 49 meV, while the measurement performed with the compressed XUV pulses provides an energy resolution of 233 meV (Figure S4b). We thus conclude that using the above-mentioned slit and pass energy, the energy resolution of the instrument is 49 meV, while in the time-resolved experiments with the compressed fs-XUV pulses the energy resolution is limited by the bandwidth of the compressed pulses. Consequently, when performing experiments with fs-XUV

radiation, using uncompressed pulses for the generation of XUV radiation is a good strategy to maximize the energy resolution in the ARPES experiments. This information is particularly helpful when performing momentum microscopy using time-of-flight spectrometers, that require a pulsed laser source.

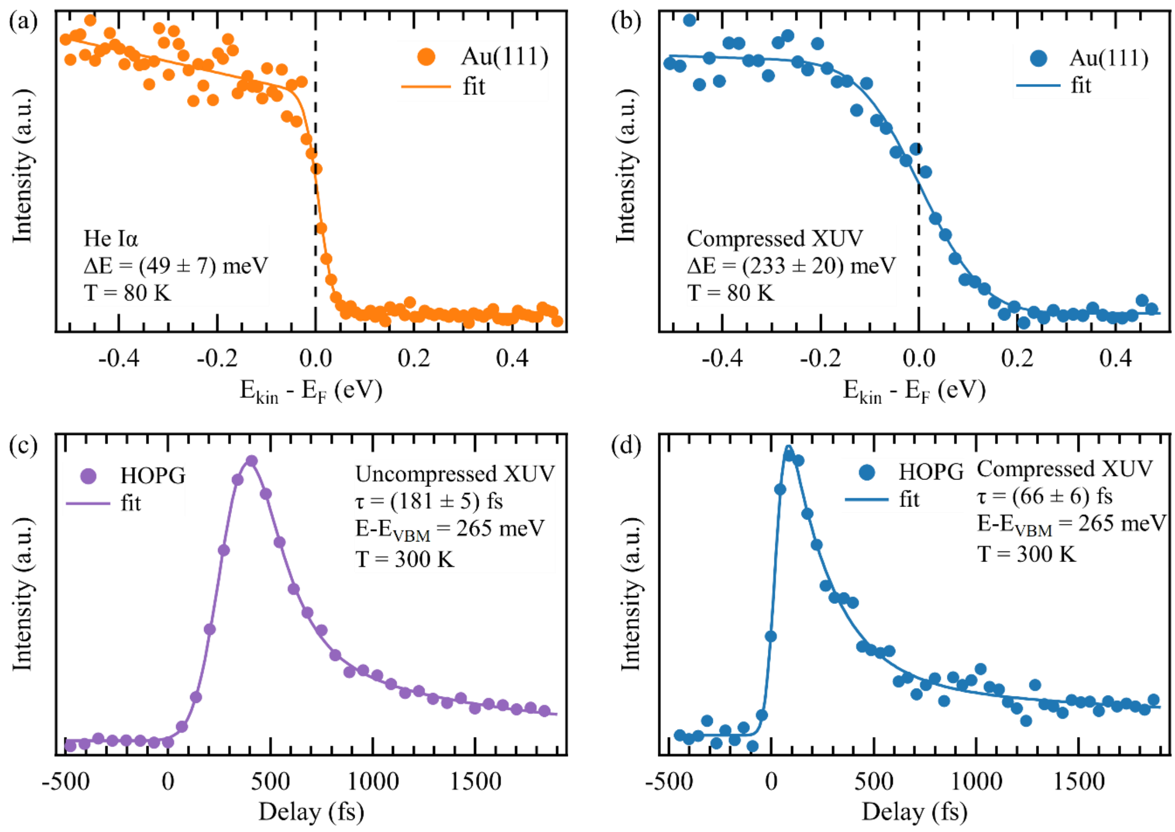

Figure S4. Comparison of energy- and time-resolution in tr-ARPES experiments. The upper two Figures show the Fermi edge of Au(111), which is fitted with a fixed Fermi-Dirac distribution convoluted with a Gaussian distribution. The full width at half maximum of the Gaussian determines the overall energy resolution, which is determined by the compressed XUV pulses in b), whereas it is limited by the entrance slit with He Iα discharge light in a). The lower Figures display transient hot electron relaxation on highly oriented pyrolytic graphite using c) uncompressed and d) compressed XUV pulses. A fit with a double exponential decay convolved with a Gaussian distribution gives time resolutions of 181 fs and 66 fs, respectively.

We now turn to the aspect of maximizing the time-resolution. Figure S4c and d display the angle-integrated photoemission intensity measured at 265 meV above the Fermi energy in a tr-ARPES experiment performed on highly oriented pyrolytic graphite (HOPG). The experiments were performed with p-polarized, frequency doubled pump pulses (photon energy 2.4 eV). The data contain information about hot electron dynamics in HOPG. We fitted the curves with a double exponential decay multiplied with a Heaviside function convoluted with a Gaussian distribution. The latter constitutes the cross-correlation of the visible pump and XUV probe pulse and can be taken as a quantitative measure for the time resolution of the experiment. Comparing the data shown in Figure S4c and d, recorded respectively

with the uncompressed and compressed pulses, the implemented nonlinear compression reduces the temporal resolution τ from (181 ± 5) fs to (66 ± 6) fs, fitting to the temporal resolution in section 4.2 of the main text within the standard deviation. We have thus proved that our experimental setup provides two distinct configuration options, selectable by just bypassing the compressor unit, offering the flexibility of varying experimental requirements focused on either temporal or energy resolution.

S4 XUV beam shape in PEEM

The second mirror of the XUV monochromator, with a 2 m radius of curvature, focuses the beam onto the sample. Figure S5 displays the beam profile at the sample position, measured on a HOPG crystal. Black line profiles along the edges represent the integrated data along each respective dimension and were fitted using a Gaussian distribution with a linear background. The resulting fits, shown as blue lines, yield a beam FWHM of 19.5 μm along the *y*-axis and 31.7 μm along the *x*-axis. The presence of white freckles throughout the image is attributed to surface impurities.

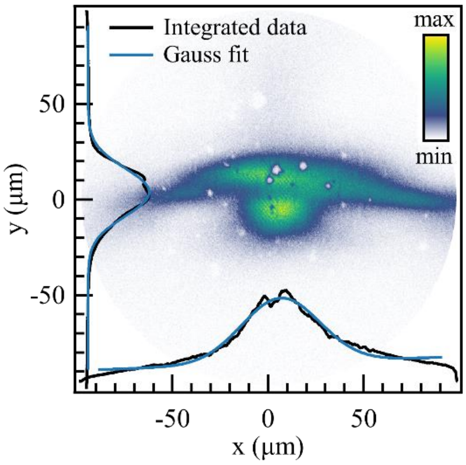

Figure S5: Beam profile in PEEM mode on HOPG. Black line profiles represent the integrated intensity along each axis of the image and were fitted with a Gaussian distribution combined with a linear background.

S5 Kreios momentum microscope resolution on the Au(111) surface state

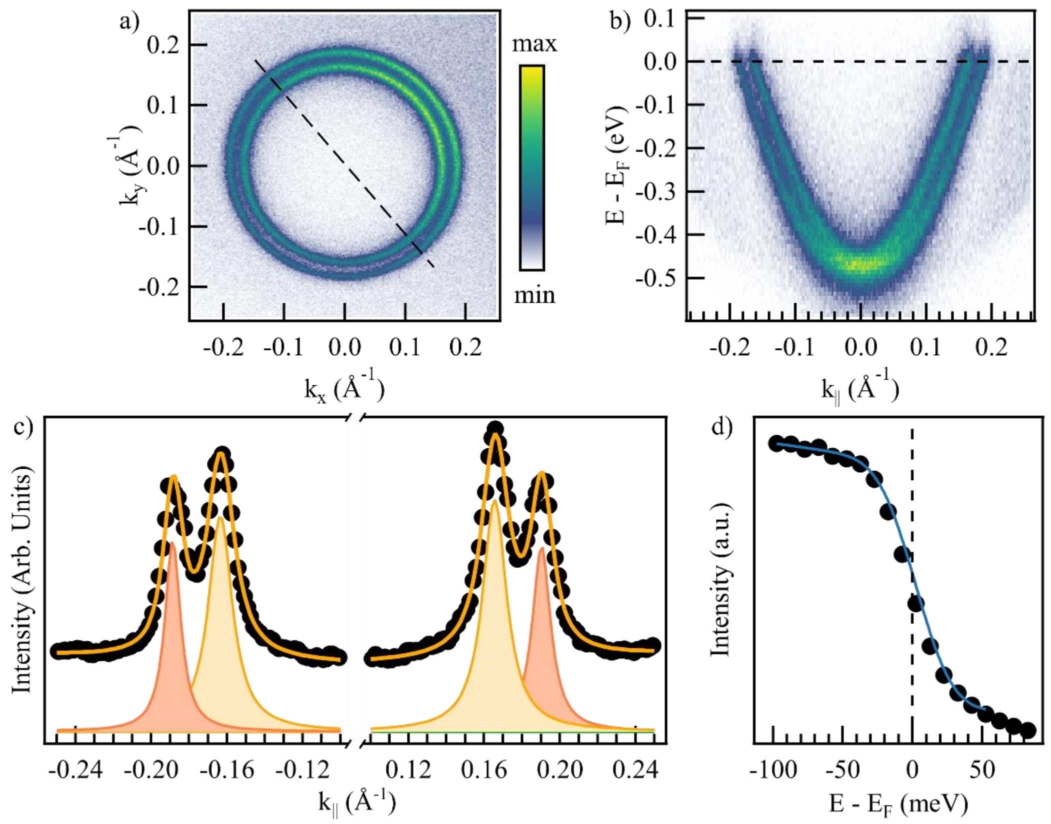

Figure S6: Surface state on Au(111) at 6.5 K. Figure a) shows a momentum map resolving the Rashba splitting. A dashed line marks the path of the band structure in b). Close to Fermi, a fit of four Voigt distributions trace the spin-split band separation and give an angular resolution of 0.0050 Å^{-1}, as presented in c). The Fermi edge in d) is fitted with a convolved Gaussian and Fermi function reading an energy resolution of 49 meV.

The resolution of the microscope in terms of energy, momentum, and real space is contingent upon the configuration of the lens system, the size of the entrance slit, and the pass energy of the hemispherical analyzer. Figure S6 illustrates a measurement conducted on the Au(111) surface state, employing optimized settings to achieve high angular and energy resolution at 6.5 K (entrance slit 0.2×0.8 mm). We use 6 eV photons to maximize both resolutions, as elaborated in detail in reference [2]. In Figure S6a, a momentum map is presented, showcasing two sub-bands of the surface state at 10 meV below the Fermi energy as two concentric rings. The Rashba splitting of the surface state is attributed to spin-orbit coupling and its dispersion is depicted in Figure S6b, consistent with prior observations [3]. A cross section along the dashed line in Figure S6a is displayed in Figure S6c. We fitted the curve with four Voigt functions sharing a common Gauss width parameter, which gives an angular resolution of 0.005 Å^{-1}. From the Lorentzian peak position, we determine an angular separation of 0.025 Å^{-1} between

the inner and outer ring, aligning perfectly with previous findings by C. Tusche et al [4]. Additionally, the energy resolution is 49 meV, determined by fitting the Fermi edge curve in Figure S6d using a convolution of a fixed Fermi distribution and a Gaussian distribution.

S6 Transient valley occupation of WS_2

We observe a strong signal in the $\overline{\mathrm{K}}$ and $\overline{\Sigma}$ conduction band valleys upon photoexcitation with a 2.4 eV pump pulse. From visual inspection, the lifetime of the electron distribution in the $\overline{\Sigma}$ valley is much greater than in the $\overline{\mathrm{K}}$ valley and also their respective rise times differ. This is in line with previous studies on hot carrier dynamics in WS_2 that have also found valley-dependent relaxation dynamics [5–7]. In bulk WS_2, electrons in the $\overline{\mathrm{K}}$ valley can either recombine directly or undergo intervalley scattering into the $\overline{\Sigma}$ valley, where subsequent recombination is indirect. Hence, we model the transient valley populations with

$$\frac{dN_K(t)}{dt} = -\left(\frac{1}{\tau_{K\to\Sigma}} + \frac{1}{\tau_K}\right) \cdot N_{K,0} \tag{S2}$$

$$\frac{\mathrm{d}N_\Sigma(t)}{\mathrm{d}t} = -\frac{1}{\tau_\Sigma} \cdot N_\Sigma(t) + \frac{1}{\tau_{K\to\Sigma}} \cdot N_{K,0}, \tag{S3}$$

where τ_K, τ_Σ, and $\tau_{K\to\Sigma}$ denote the three described relaxation rates. We fit the curves depicted in the main text (compare Figure 6c) with a convolution of a Gaussian distribution and the respective relaxation function multiplied with the Heaviside function, reading

$$I_\mathrm{K}(t) = \int \frac{N_{K,0}}{\sqrt{2\pi\sigma^2}} \times \exp\left(-\frac{(t'-\mu)^2}{2\sigma^2}\right) \times \exp\left(-(t-t')\left(\frac{1}{\tau_K} + \frac{1}{\tau_{K\to\Sigma}}\right)\right) \times \Theta(t-t')\,\mathrm{d}t' \tag{S4}$$

$$I_\Sigma(t) = \int \frac{N_{\Sigma,0}}{\sqrt{2\pi\sigma^2}} \times \exp\left(-\frac{(t'-\mu)^2}{2\sigma^2}\right) \times \left(\exp\left(-\frac{t-t'}{\tau_{\Sigma\to K}}\right) - \exp\left(-\frac{t-t'}{\tau_\Sigma}\right)\right) \times \Theta(t-t')\,\mathrm{d}t' \tag{S5}$$

S7 References

1. J. Weitenberg, A. Vernaleken, J. Schulte, et al., "Multi-pass-cell-based nonlinear pulse compression to 115 fs at 7.5 µJ pulse energy and 300 W average power," Opt. Express **25**, 20502–20510 (2017).
2. S. Ponzoni, F. Paßlack, M. Stupar, et al., "Dirac Bands in the Topological Insulator Bi_2Se_3 Mapped by Time-Resolved Momentum Microscopy," Adv. Phys. Res. **2**, 2200016 (2023).

3. S. LaShell, B. A. McDougall, and E. Jensen, "Spin Splitting of an Au(111) Surface State Band Observed with Angle Resolved Photoelectron Spectroscopy," Phys. Rev. Lett. **77**, 3419–3422 (1996).

4. C. Tusche, A. Krasyuk, and J. Kirschner, "Spin resolved bandstructure imaging with a high resolution momentum microscope," Ultramicroscopy **159**, 520–529 (2015).

5. F. Liu, Q. Li, and X.-Y. Zhu, "Direct determination of momentum-resolved electron transfer in the photoexcited van der Waals heterobilayer WS_2/MoS_2," Phys. Rev. B **101**, 201405 (2020).

6. R. Wallauer, R. Perea-Causin, L. Münster, et al., "Momentum-Resolved Observation of Exciton Formation Dynamics in Monolayer WS_2," Nano Lett. **21**, 5867–5873 (2021).

7. Y. Zhu, O. V. Prezhdo, R. Long, et al., "Twist Angle-Dependent Intervalley Charge Carrier Transfer and Recombination in Bilayer WS_2," J. Am. Chem. Soc. **145**, 22826–22835 (2023).
